# A Rare Case of Brachyolmia with Amelogenesis Imperfecta Caused by a New Pathogenic Splicing Variant in *LTBP3*

**DOI:** 10.3390/genes12091406

**Published:** 2021-09-12

**Authors:** Elisabetta Flex, Valentina Imperatore, Giovanna Carpentieri, Alessandro Bruselles, Andrea Ciolfi, Simone Pizzi, Maria Giovanna Tedesco, Daniela Rogaia, Amedea Mencarelli, Giuseppe Di Cara, Alberto Verrotti, Stefania Troiani, Giuseppe Merla, Marco Tartaglia, Paolo Prontera

**Affiliations:** 1 Department of Oncology and Molecular Medicine, Istituto Superiore di Sanità, 00161 Rome, Italy; elisabetta.flex@iss.it (E.F.); giovanna.carpentieri@iss.it (G.C.); alessandro.bruselles@iss.it (A.B.); 2 Medical Genetics Unit, University and Hospital of Perugia, 06129 Perugia, Italy; valentina.imperatore@ospedale.perugia.it (V.I.); tedesco.mariag@libero.it (M.G.T.); daniela.rogaia@ospedale.perugia.it (D.R.); amedea.mencarelli@ospedale.perugia.it (A.M.); 3 Genetics and Rare Diseases Research Division, Ospedale Pediatrico Bambino Gesù, IRCCS, 00146 Rome, Italy; andrea.ciolfi@opbg.net (A.C.); simone.pizzi@opbg.net (S.P.); marco.tartaglia@opbg.net (M.T.); 4 Pediatric Clinic, University and Hospital of Perugia, 06129 Perugia, Italy; giuseppe.dicara@unipg.it (G.D.C.); alberto.verrotti@ospedale.perugia.it (A.V.); 5 Neonatal Intensive Care Unit, University and Hospital of Perugia, 06129 Perugia, Italy; stefania.troiani@ospedale.perugia.it; 6 Department of Molecular Medicine and Medical Biotechnology, University of Naples Federico II, 80131 Naples, Italy; giuseppe.merla@unina.it

**Keywords:** brachyolmia, amelogenesis imperfecta, *LTBP3*, whole exome sequencing, consanguinity

## Abstract

In recent years, a rare form of autosomal recessive brachyolmia associated with amelogenesis imperfecta (AI) has been described as a novel nosologic entity. This disorder is characterized by skeletal dysplasia (e.g., platyspondyly, short trunk, scoliosis, broad ilia, elongated femoral necks with coxa valga) and severe enamel and dental anomalies. Pathogenic variants in the latent transforming growth factor-β binding protein 3 (*LTBP3*) gene have been found implicated in the pathogenesis of this disorder. So far, biallelic pathogenic *LTBP3* variants have been identified in less than 10 families. We here report a young boy born from consanguineous parents with a complex phenotype including skeletal dysplasia associated with aortic stenosis, hypertrophic cardiomyopathy, hypodontia and amelogenesis imperfecta caused by a previously unreported homozygous *LTBP3* splice site variant. We also compare the genotypes and phenotypes of patients reported to date. This work provides further evidence that brachyolmia with amelogenesis imperfecta is a distinct nosologic entity and that variations in *LTBP3* are involved in its pathogenesis.

## 1. Introduction

Brachyolmia constitutes a clinically and genetically heterogeneous group of rare bone disorders characterized by short stature, short trunk, scoliosis, and platyspondyly (OrphaNumber: 1293). Several types of brachyolmia have been described based on clinical criteria and inheritance model [1]. Currently, four major forms of brachyolmia have been recognized, including the Hobaek (OMIM 271530) and Toledo (OMIM 271630), Maroteaux (OMIM 613678), dominant (OMIM 113500), and brachyolmia with mild epiphyseal and methaphyseal changes (OMIM 612847) types. The first two forms together with type 4 are characterized by autosomal recessive inheritance associated with biallelic variations in the *PAPSS2* gene (10q23.2q23.31) [2]. Both Hobaek and Toledo forms are characterized by scoliosis, platyspondyly with elongated vertebral bodies, overfaced pedicles and irregular, narrow invertebral spaces, with the latter showing corneal opacities and precocious calcification of the costal cartilagine. The Maroteaux type is distinguished by the rounded form of the vertebral borders with shorter vertebral bodies compared to Hobaek and Toledo type. The dominant form is a very rare skeletal dysplasia caused by pathogenic variants in the *TRPV4* gene. This condition is characterized by severe kyphoscoliosis and flattened, irregular cervical vertebrae [3]. The diagnosis of brachyolmia is achieved by clinical and radiological findings; molecular genetic testing is useful to reach a precise diagnosis. To date, no specific treatment is available for this disorder, but the prognosis for patients with brachyolmia is generally good.

Amelogenesis imperfecta (AI) is a group of rare heterogeneous inherited disorders characterized by defective or missing tooth enamel, affecting all or nearly all the teeth. In particular, the enamel appears hypoplastic and/or hypomineralized and the affected teeth, which are discolored and sensitive, often tend to disintegration. AI, whose prevalence is estimated between 1:700 and 1:14,000, is commonly associated with morphological abnormalities in other organs [4]. This disorder shows autosomal dominant, autosomal recessive and X-linked inheritance patterns. Variants in the amelogenin gene, *AMELX* (Xp22.3-p22.1, OMIM *300391), underlie the X-linked form of AI [5], while pathogenic variants in the enamelin gene, *ENAM* (4q21, OMIM *606585), are implicated in the development of autosomal dominant and autosomal recessive conditions [6].

Dental anomalies and short stature (DASS, OMIM #601216) was reported as a distinct entity by *Verloes A* et al. in 1996. They described a previously unrecognized autosomal recessive form of skeletal dysplasia with platyspondyly and AI in two siblings from a consanguineous family. These children showed AI in association with short trunk and brachyolmia-like anomalies, including platyspondyly, narrowed intervertebral and interpedicular distances, rectangular-shaped vertebrae with posterior scalloping, herniation of the nuclei and broad femoral necks [7]. In 2009, *Bertola* et al. [8] confirmed that skeletal (brachyolmia) and dental (AI) abnormalities are not a cooccurring association but rather a specific condition. Indeed, recent studies demonstrated that pathogenic variants in the latent transforming growth factor (TGF)-β binding protein 3 gene (*LTBP3*, 11q13.1, OMIM *602090) are responsible for brachyolmia with different teeth disorders [9,10,11].

More recently, two other unrelated families with DASS and thoracic aortic aneurysm associated with pathogenic variants in *LTBP3* have been described by *Guo* et al. [12]. Notably, pathogenic variations in *LTBP3* have also been associated with acromic dysplasia (ACMICD, OMIM #102370) and geleophysic dysplasia 3 (GPHYSD3, OMIM #617809). ACMICD and GPHYSD3 are characterized by short stature, brachydactyly, delayed bone age and progressive joint limitation [13,14]. Additionally, individuals with GPHYSD3 also present progressive cardiac and respiratory problems. Both disorders do not include tooth involvement.

To date, only three and two monoallelic pathogenic *LTBP3* variants have been identified in ACMICD [15] and GPHYSD3 [16] respectively, while 11 biallelic *LTBP3* variants have been associated with DASS [9,10,11,12,15,17].

We report on an affected boy, born from consanguineous parents with negative family history, showing DASS phenotype (brachyolmia with AI, hereafter) associated to a previously unreported homozygous splicing variant (c.2894-2A>G) in the *LTBP3* gene. This finding further confirms the specific association of skeletal and dental abnormalities as major features characterizing this clinical entity.

## 2. Patient

The proband was a 14-year-old boy born in Perù by natural delivery from first cousin consanguineous parents, at the end of an uneventful pregnancy.

**Figure 1:**
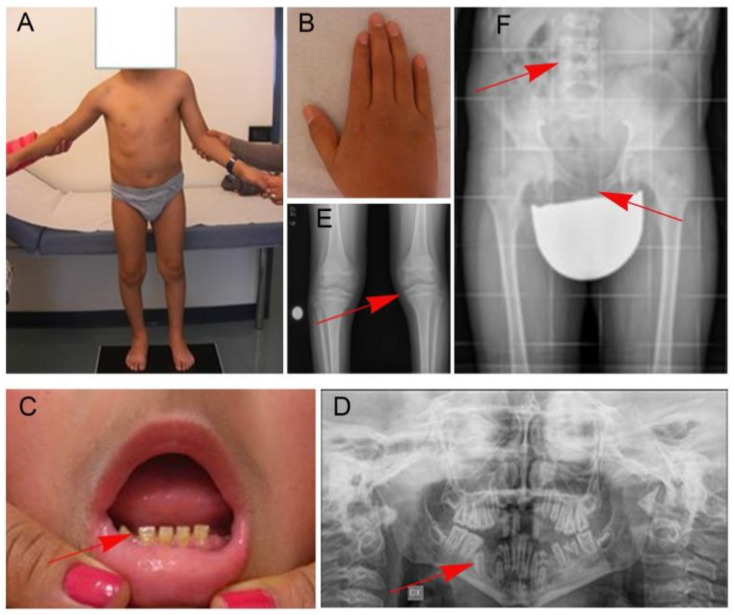
**Clinical features of the subject with the homozygous *LTBP3* splice site variant.** Proband’s photographs showing short trunk, varus knees, upper limbs diplegia (**A**) and brachydactyly (**B**). The subject also showed amelogenesis imperfecta and hypodontia (**C**). Radiographic examinations of the dental archs, lower limbs, pelvis and trunk demonstrated oligodontia (red arrow in panel (**D**), slightly widened appearance of the proximal epiphyseal region of the tibiae and of the distal epiphyseal region of the femurs (**E**), platyspondyly (**F**).

Birth weight was 3060 g and length was 52 cm. First words were regularly achieved and he started to walk independently at 12 months. Psychomotor development and growth were normal up to the age of 3 years, when he started to present a slowdown in growth and a progressive decrease of motor abilities. At the time of the first evaluation, the patient was nine years old and a diagnosis of skeletal dysplasia with aortic stenosis and hypertrophic cardiomyopathy was made. Since 2012, multiple syncopal attacks occurred. In 2015, severe lower limbs diplegia was observed. Metabolic analyses were normal. At the time of our genetic counselling (14 years), the proband showed short stature, short trunk, scoliosis with accentuated lordosis, facial dysmorphisms, pterygium colli, varus knees, lower limbs diplegia and platyspondyly (Figure 1A,B). Dental evaluations revealed hypodontia and amelogenesis imperfecta (Figure 1C,D). Radiographic examinations of upper limbs and pelvis disclosed slightly widened appearance of the proximal epiphyseal region of the tibiae and of the distal epiphyseal region of the femurs (Figure 1E,F).

The boy had difficulties in standing and walking. Brain magnetic resonance imaging (MRI) showed no relevant anomalies. He had normal intelligence. Karyotype and array-CGH analyses were normal.

## 3. Materials and Methods

### 3.1. Samples and RNA/DNA Extraction

The patient was followed at the Medical Genetics Unit of Azienda Ospedaliera of Perugia. Genomic DNA, clinical data and photographs were collected, used and analyzed for both diagnosis and research purpose after written informed consent was provided and signed by the mother (the only legal tutor of the child). Genomic DNA of the proband and his mother were isolated from EDTA peripheral blood sample using QIAmp DNA Blood Kit according to the manifacturer’s protocol (Qiagen, Hilden, Germany). RNA was isolated from whole blood stabilized in PAXgene Blood RNA tubes (PreAnalytiX, Qiagen/BD Company, Hombrechtikon, Switzerland). Peripheral blood mononuclear cells (PBMC) were isolated by density-gradient centrifugation using media Ficoll-Paque (SIGMA-Aldrich, Merck KGaA, Darmstadt, Germania), and total RNA was extracted by the RNeasy Mini Kit (Qiagen), following manufacturer’s protocol.

### 3.2. Comparative Genomic Hybridization Analysis

The array-comparative genomic hybridization (array-CGH) analysis was performed using InnoScan710 platform (InnoPSYS, Carbonne, France). The experiment was carried out on a CytoChip ISCA 4 × 180 K v.1.0 (BlueGnome, Cambridge, UK). Graphical visualization of results was obtained by the BluFuse Multi v.3.0 Software (Illumina, San Diego, CA, USA).

### 3.3. Whole Exome Sequencing

Exome sequencing was performed in the frame of the Ospedale Pediatrico Bambino Gesù’s “Undiagnosed Patients Program”. The library preparation was carried out using the NimbleGen SeqCap EZ V.2 (Roche, Madison, WI, USA) on DNA obtained from leukocytes. Parallel sequencing was performed using a HiSeq2000 platform (Illumina). Sequencing generated 135 million high-quality reads (i.e., >75% of bases having Q30), obtaining 86x effective mean target coverage depth, and more than 94% of bases covered ≥20x. Data analysis was carried out using an in-house implemented pipeline, which mainly took advantage of the Genome Analysis Toolkit (GATK V.3.7) [18] framework, as previously reported [19,20,21,22]. Reads mapping on human genome assembly GRCh37 (hg19) was performed by BWA V.0.7.12 [23], and GATK tools were used for base quality recalibration and variants calling. SNVs and small INDELs were identified by means of the GATK’s HaplotypeCaller. Finally, variants were quality-filtered using a hard-filters strategy, according to GATK’s 2016 best practices. High-quality variants were then filtered against public databases (dbSNP150 and gnomAD V.2.1) to retain private and clinically associated variants, annotated variants with unknown frequency or having MAF < 1% and occurring with a frequency < 1% in an in-house database including frequency data from approximately 2000 population-matched WES. SnpEff toolbox (V.4.3) and dbNSFP (V.3.5) were used to predict the functional impact of variants to retain only those located in exons with any effect on the coding sequence and splice site regions (variants located from −3 to +8 with respect to an exon-intron junction) [24,25,26]. Predicted functional impact of variants was accessed by Combined Annotation Dependent Depletion (CADD) V.1.4, M-CAP V.1.0, and InterVar, V.2.0.1 algorithms [27,28,29] to obtain clinical interpretation according to ACMG/AMP 2015 guidelines [29]. Variants were prioritized on the basis of the functional relevance of genes, considering recessive (homozygosity by descent) and X-linked inheritance models. WES statistics are reported in Supplementary Table S1. Variant validation and segregation were attained by Sanger sequencing.

### 3.4. Sanger Sequencing

The variants of interest were validated by Sanger sequencing using an ABI 3500 Genetic Analyzer (Applied Biosystems, Foster City, CA, USA) and the ABI BigDye Terminator Sequencing Kit, V.3.1 (Applied Biosystems). Primers are available upon request. All variants are described according to Human Genome Variations Society (HGVS) [30]. Nucleotide numbers are derived from the cDNA sequence of *LTBP3* (GenBank accession no.NM_001130144.2, genome version GRCh37/hg19).

### 3.5. RNA Analysis

RNA was retro-transcribed using SuperScriptIII first strand kit (Invitrogen, Carlsbad, CA, USA), following the manufacturer’s instructions. RT-PCR was carried out using primers specifically designed to amplify exons 20–22 (forward: 5′-CCACCACAAGAAGGAGTGC-3′; reverse: 5′-GTAGAAGCCCTGCTTGCAG). The housekeeping *GAPDH* mRNA was used as control.

## 4. Results

### 4.1. WES Analysis

Exome analysis was performed on DNA extracted from leukocytes of the proband. Data analysis, variant filtering and prioritization compatible with either a recessive (homozygosity by descent) or X-linked transmission model allowed for the identification of a private homozygous splice site variant (c.2894-2A>G) in *LTBP3* as the event likely underlying the disorder (Table 1). The variant had not previously been reported in public and in-house databases and was considered as damaging by in silico prediction tools (CADD v1.4 score = 27.2, spliceAI score = 0.97, dbscSNV score = 0.92).

**Figure 2:**
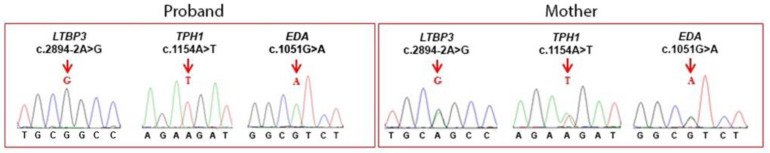
**Sanger validation and variants segregation.** The figure shows Sanger sequence chromatograms of the selected genes in the proband and his mother. Nucleotide numbers are derived from the coding sequence of *LTBP3* (GenBank accession NM_001130144.2), *TPH1* (GenBank accession NM_004179) and *EDA* (GenBank accession NM_001399).

The variants were confirmed by Sanger sequencing in both proband and his mother (Figure 2).

### 4.2. RNA Analysis

To validate the functional and clinical relevance of the *LTBP3* variant, the consequence of the c. 2894-2A>G substitution on *LTBP3* transcript processing was assessed by direct sequencing of the relevant portion of the *LTBP3* cDNA obtained from total RNA extracted from peripheral blood mononuclear cells.

**Figure 3:**
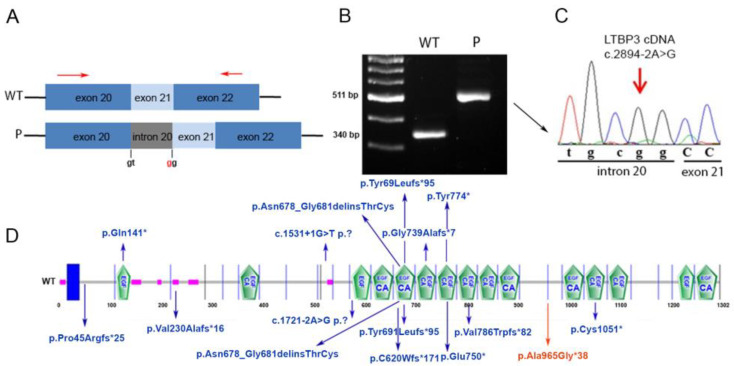
Characterization of the effect of the identified splicing variant c.2894-2A>G. (**A**) Schematic representation of the portion of the *LTBP3* coding sequence encompassing exons 20–22. The c.2894-2A>G variant is located at the exon 21 acceptor splice site, promoting retention of intron 20. Arrows above the exons indicate the primers used for the cDNA analysis. (**B**) Gel electrophoresis analysis of the amplified cDNA stretch encompassing exon 21 showing an aberrant transcript size (511 bp) in the proband (P) indicating retention of intron 20. The length of a properly processed transcript is also shown for comparison (WT). The proband’s band was purified and Sanger sequenced. (**C**) chromatogram showing the aberrant intron 20 retention. (**D**) Schematic diagram representation of the domain structure of the LTBP3 protein (NP_001123616.1) using the Simple Modular Architecture Research Tool (SMART: http://smart.embl.heidelberg.de, accessed on 1 December 2020) (above). In blue, the position of the previously identified homozygous pathogenic variants associated with DASS is shown. The presently identified pathogenic variation is shown in red.

As shown in Figure 3, the disease-associated variant altered proper splicing of the *LTBP3* transcript, leading to retention of intron 20 and, in turn, to premature truncation of translation, predicting a shorter protein lacking the functionally relevant domains at the C-terminus (http://smart.embl.heidelberg.de, accessed on 1 December 2020).

## 5. Discussion

In this study, we report on a patient with brachyolmia-AI caused by a novel homozygous inactivating variant in *LTBP3*. mRNA analysis demonstrated the aberrant processing of the transcrtipt, which was predicted to result in a truncated protein missing a functionally relevant portion at the C-terminus of the protein.

Homozygosity for the splice site change leading to premature termination is in line with the previously collected data on disease-causing *LTBP3* variants. Biallelic variants in *LTBP3* have previously been reported in brachyolmia-AI, the majority representing frameshift and nonsense variants.

LTBPs are a group of extracellular multi-domain proteins with several biological activities. These proteins are known to form a large latent complex with TGFβ and its propeptide (latency associated peptide, LAP) [31], called “latent TGFβ binding proteins”. LTBPs are required for targeting and activation of TGFβ. LTBP3, which shares a similar structure to human fibrillin-1, is coexpressed with TGFβ [32]. Binding of TGFβ to LAP is necessary to the LTBP3 secretion suggesting that it is unlikely that LTBP3 is involved in TGFβ-independent functions [33,34]. Molecular characterization of a generated Ltbp3 KO mouse demonstrated that LTBP3 regulates the bioavailability of TGFβ in chondrocytes [35], and *Ltbp3* null mice are more than 50% smaller than *wild-type* mice [36]. LTBP3 facilitates TGFβ secretion [37,38] and its localization to specific sites in the extracellular matrix [39], playing a crucial role in skeletal formation as well as tooth development [33,40].

In 2009, *Noor* et al. reported the first study in which *LTBP3* variations were associated with a human disease [9]. More recently, pathogenic variants in *LTBP3* have been found to be implicated in the pathogenesis of brachyolmia-AI in a small number of unrelated families [10,11,12,15,17].

To the best of our knowledge, previously reported patients did not show syncopal episodes, muscle weakness and decreased motor ability/diplegia as associated features. Careful clinical assessment of additional patients carrying biallelic inactivating variants in *LTBP3* is required to confirm a causal association between *LTBP3* loss of function and the additional features documented in the present patient. While it is plausible that additional variants might contribute to the complexity of the phenotype, WES/CGH array analysis did not allow for the identification of any additional *bona fide* pathogenic variant related with the additional features observed in our patient. Besides the *LTBP3* variant, however, a homozygous missense change in *TPH1* (c.1154A>T, p.Lys385Met) not reported in public databases, and a rare hemizygous substitution in *EDA* (c.1051G>A, p.Val351Ile), reported in dbSNP (rs758752553), were identified as additional functionally relevant variants. *TPH1* encodes for tryptophan hydroxylase 1, an enzyme that catalyzes the first and rate-limiting step in the biosynthesis of the neurotransmitters serotonin and melatonin [41]. Polymorphisms in *TPH1* have been associated with different neurologic disorders, including schizophrenia [42] and suicidal behavior [43]. Our patient did not suffer from psychiatric disorders; however, the role of *TPH1* variations in human diseases in still not completely understood and we cannot exclude that some clinical aspect of the child (i.e., diplegia, a feature not typical of brachiolmia-AI) may be associated to this specific variant. Similarly, variations in *EDA* are associated with hypohidrotic, or anhidrotic, ectodermal dysplasia, a disorder characterized by a triad of signs comprising: sparse hair (hypotrichosis), abnormal or missing teeth (anodontia or hypodontia) and inability to sweat (anhidrosis or hypohidrosis). Typical clinical manifestations also include dryness of the skin, eyes, airways and mucous membranes presumably due to the defective development of several exocrine glands, which had not been documented in the present patient. The occurrence of hypodontia cannot be considered as sufficient for a diagnosis of hypohydrotic ectodermal dysplasia and represents a key feature in patients with pathogenic biallelic *LTBP3* variants. Furthermore, a non-syndromic familial hypodontia (NHS) is associated with pathogenic variants in *EDA* that occurs either sporadically or as familial trait [44]. Probands affected by these variations show only variable degrees of tooth lack, incisors, molar and premolar mainly (without other signs associated with ectodermal dysplasia) [45]. We cannot exclude a potential role of *EDA* variant in the etiopathogenesis of hypodontia in our patient, but without a positive family history and with his mother being healthy, it appears more likely that the dental anomalies, along with other symptoms, were caused by *LTBP3* variant. Future literature’s genotype-phenotype studies on *EDA* can solve this pending question.

A comprehensive summary of all reported pathogenic *LTBP3* variants is shown in Table 2.

Genotype-phenotype correlations between the different clinical features characterizing ACMICD, GPHYSD3 and DASS, and the location and type of the underlying pathogenic variation have not been reported. It is apparent that the observed clinical variability within *LTBP3* variants may be attributable both to the varying nature of the pathogenic variation, which involves the C-terminal domain of the protein (involving the epidermal growth factor (EGF) like calcium-binding domain) in most cases and the different inheritance pattern. While biallelic loss-of-functions variants are reported in DASS, at least one heterozygous stop-loss and one splice site variant have been described in patients with GPHYSD3 as well as heterozygous gain-of-function missense variants that have been associated with ACMICD. The functional roles of the heterozygous stop-loss and the splice site variants identified in patients with GPHYSD3 were not investigated, their gain or loss-of-function effects remain therefore to be elucidated. Consistently, ACMICD and GPHYSD3 are characterized by a more similar phenotype compared to DASS and have been considered entities belonging to the same clinical spectrum [14].

A genotype-phenotype correlation study has been made by *Intarak.* and by *Kaur.* [15,17], where bi-allelic loss-of-function variations were found mainly in DASS, and monoallelic missense gain of function or dominant negative pathogenic variants in the highly conserved EGF-like calcium-binding domain were associated with ACMID and monoallelic stop-loss or splicing variants with GPHYSD3. Starting from this and adding our and last *LTBP3* studies (Table 2) we were not able to find other peculiar association between variants and phenotypes.

In conclusion, the present finding further supports the evidence that inactivating biallelic *LTBP3* variants are responsible for a very rare, autosomal recessive disorder, named brachyolmia with AI. The clinical significance of the *TPH1* variant in the pathogenesis of the neurological phenotype in our patient deserves attention. Additional unrelated cases sharing variants in the *TPH1* gene and similar neuromuscular features might be useful in validating this possible association.

## Figures and Tables

**Table 1:** Identified variants by WES analysis.

	Location	Gene	Transcript	Variation	Protein Level	Note
**Homozygous**	chr11:65308427	*LTBP3*	NM_001130144.3	c.2894-2A>G	p.Ala965Glyfs*38	Latent transforming growth factor (TGF) beta binding protein
	chr11:18044351	*TPH1*	NM_004179.3	c.1154A>T	p. Lys385Met	Tryptophan hydroxylase 1
**Hemizygous**	chrX:69255334	*EDA*	NM_001399.5	c.1051G>A	p.Val351Ile	Ectodysplasin A

**Legend.** The table reports the most clinically/functionally relevant homozygous variants identified by exome analyses. Coordinates are according to hg19.

**Table 2:** Clinical phenotype of brachyolmia with amelogenesis imperfecta (AI) and identified disease-causing variants reported in literature.

			*Noor* et al., *(2009)*	*Huckert* et al., *(2015)*					*Dugan* et al., *(2015)*	
***General Information***		Age (y/m)	30y ^a^	14y	13y ^b^	13y ^b^	11y	16y ^c^,9y ^c^,12y ^c^	18y ^d^	15y ^d^
		Gender (M/F)	M	F	F	M	M	F,F,M	F	F
		Number of affected individuals studied in the family	4	2	2		1	3	2	
		Ethnic	Punjabi	Turkey	Caucasian French		Brazil	Pakistan	Emirati	
		Parental conseguinity	Yes	Yes	No		Yes	Yes	No	
Clinical Features	***Growth***	Normal birth length	NA	NA	NA	NA	NA	NA	−	+
		Short stature	+	+	+	+	+	+	+	+
		Short neck	NA	NA	NA	NA	NA	NA	NA	NA
		Short trunk	NA	NA	NA	NA	NA	NA	NA	NA
	**Eyes**	Corneal Opacities	NA	NA	NA	NA	NA	NA	NA	NA
		Myopia	NA	+	−	−	NA	NA	NA	NA
		Hyperopia	NA	NA	NA	NA	NA	NA	NA	NA
	**Teeth**	Retarded teeth eruption	NA	NA	NA	NA	+	NA	+	+
		Amelogenesis imperfecta	+	+	+	+	+	+	NA	NA
		Oligodotia	+	+	NA	NA	−	+	+	+
	**Skeletal**	Pectus carinatum	NA	NA	NA	+	NA	NA	NA	NA
		Osteopenia	−	+	NA	NA	NA	+	NA	NA
		Platyspondyly	−	+	+	NA	+	+	NA	NA
		Short pedicles	NA	NA	NA	NA	NA	NA	+	+
		Vertebral borders rounded anteriorly and posteriorly	NA	NA	NA	NA	NA	NA	NA	NA
		Irregular end plates							+	+
		Narrow intervertebral spaces	NA	NA	NA	NA	NA	NA	+	+
		Scoliosis	+	+	NA	NA	+	+	+	+
		Kyphosis	NA	NA	NA	NA	NA	NA	+	+
		Gibbus	NA	NA	NA	NA	NA	NA	NA	NA
		Short femoral neck	NA	NA	NA	NA	NA	NA	NA	NA
		Irregular femoral metaphyses	NA	NA	NA	NA	NA	NA	NA	NA
		Short iliac bones	NA	NA	NA	NA	NA	NA	NA	NA
		Irregular epiphyses	NA	NA	NA	NA	NA	NA	NA	NA
		Short and bowed lower limbs	NA	NA	NA	NA	NA	NA	NA	NA
		Enlarged knee joints	NA	NA	NA	NA	NA	NA	NA	NA
		Metaphyseal changes of knees and hips	NA	NA	NA	NA	NA	NA	NA	NA
		Osteoarthropathy, precocious	NA	NA	NA	NA	NA	NA	NA	NA
		Slightly short long bones	NA	NA	NA	NA	NA	NA	NA	NA
	**Skin, Hands & Hair**	Brachydactyly	−	−	+	+	NA	NA	±	±
		Arachnodactyly	NA	NA	NA	NA	NA	NA	+	−
		Acne	NA	NA	NA	NA	NA	NA	−	−
		Acanthosis nigricans	NA	NA	NA	NA	NA	NA	+	−
		Hirsutism	NA	NA	NA	NA	NA	NA	NA	NA
	**Neurologic**	Normal intelligence	+	NA	NA	NA	NA	NA	+	−
		Spinal cord compression	NA	NA	NA	NA	NA	NA	NA	NA
		Falx cerebri, precocious calcification of	NA	NA	NA	NA	NA	NA	NA	NA
	**Cardiac**	Thoracic aortic aneurysms/dissections	NA	NA	NA	NA	NA	NA	NA	NA
		Mild mitral valve prolapse	NA	NA	NA	NA	NA	NA	+	+
		Cardiomyopathy	NA	−	−	−	NA	NA	+	+
Molecular Finding		**Inheritance patterns**	AR	AR	AR*	AR*	AR	AR	AR	AR
		**Gene**	*LTBP3*	*LTBP3*	*LTBP3*	*LTBP3*	*LTBP3*	*LTBP3*	*LTBP3*	*LTBP3*
		**Exon**	16	14	2/8	2/8	15	17	13	13
		**DNA**	c.2322C>G	c.2071_2084delTAC CGG CTC AAA GC	c.421C>T; c.1531+1G>T	c.421C>T; c.1531+1G>T	c.2216_2217delG	c.2356_2357delG	c.1858_1859delG	c.1858_1859delG
		**Protein**	p.Tyr744*	p.Tyr691Leufs*95	p.Gln141*; p.(?)	p.Gln141*; p.(?)	p.Gly739Alafs*7	p.Val786Trpfs*82	p.Cys620Trpfs*171	p.Cys620Trpfs*171
			***Guo* et al., *(2018)***					***Intarak* et al., *(2019)***	***Kaur* et al., *(2020)***	***Present Study***
***General Information***		Age (y/m)	54y ^e^	55y ^e^	59y ^e^	44y ^f^	58y ^f^	24y ^g^	7y	14y
		Gender (M/F)	M	F	F	M	F	M	F	M
		Number of affected individuals studied in the family	3			2		1	1	1
		Ethnic	American			American		Thai	India	Perù
		Parental conseguinity	No			Yes		Yes	No	Yes
Clinical Features	**Growth**	Normal birth length	NA	NA	NA	NA	NA	NA	+	+
		Short stature	+	+	+	+	+	+	+	+
		Short neck	NA	NA	NA	NA	NA	NA	NA	NA
		Short trunk	NA	NA	NA	NA	NA	NA	+	+
	**Eyes**	Corneal Opacities	NA	NA	NA	NA	NA	NA	NA	−
		Myopia	NA	NA	NA	NA	NA	−	NA	−
		Hyperopia	NA	NA	NA	NA	NA	NA	NA	NA
	**Teeth**	Retarded teeth eruption	NA	NA	NA	NA	NA	−	+	NA
		Amelogenesis imperfecta	+	+	+	+	+	+	+	+
		Oligodotia	NA	NA	NA	NA	NA	−	+	+
	**Skeletal**	Pectus carinatum	NA	NA	NA	NA	NA	−	NA	−
		Osteopenia	−	−	+	−	+	NA	−	−
		Platyspondyly	NA	NA	NA	NA	NA	NA	+	+
		Short pedicles	NA	NA	NA	NA	NA	NA	NA	
		Vertebral borders rounded anteriorly and posteriorly	NA	NA	NA	NA	NA	NA	NA	−
		Irregular end plates	NA	NA	NA	NA	NA	NA	NA	NA
		Narrow intervertebral spaces	NA	NA	NA	NA	NA	NA	NA	NA
		Scoliosis	−	−	+	−	+	+	−	+
		Kyphosis	NA	NA	NA	NA	NA	NA	NA	−
		Gibbus	NA	NA	NA	NA	NA	NA	NA	−
		Short femoral neck	NA	NA	NA	NA	NA	NA	NA	NA
		Irregular femoral metaphyses	NA	NA	NA	NA	NA	NA	NA	+
		Short iliac bones	NA	NA	NA	NA	NA	NA	NA	NA
		Irregular epiphyses	NA	NA	NA	NA	NA	NA	+	+
		Short and bowed lower limbs	NA	NA	NA	NA	NA	NA	NA	NA
		Enlarged knee joints	NA	NA	NA	NA	NA	NA	NA	NA
		Metaphyseal changes of knees and hips	NA	NA	NA	NA	NA	NA	NA	NA
		Osteoarthropathy, precocious	NA	NA	NA	NA	NA	NA	NA	NA
		Slightly short long bones	NA	NA	NA	NA	NA	NA	+	NA
	**Skin, Hands & hair**	Brachydactyly	NA	NA	NA	NA	NA	NA	+	+
		Arachnodactyly	NA	NA	NA	NA	NA	NA	−	−
		Acne	NA	NA	NA	NA	NA	NA	NA	−
		Acanthosis nigricans	NA	NA	NA	NA	NA	NA	NA	−
		Hirsutism	NA	NA	NA	NA	NA	NA	NA	−
	**Neurologic**	Normal intelligence	NA	NA	NA	NA	NA	NA	NA	+
		Spinal cord compression	NA	NA	NA	NA	NA	NA	NA	NA
		Falx cerebri, precocious calcification of	NA	NA	NA	NA	NA	NA	NA	NA
	**Cardiac**	Thoracic aortic aneurysms/dissections	+	+	−	+	+	−	−	NA
		Mild mitral valve prolapse	−	+	+	−	+	?	−	NA
		Cardiomyopathy	NA	NA	NA	NA	NA	NA	−	+
Molecular Finding		**Inheritance patterns**	AR*	AR*	AR*	AR	AR	AR	AR*	AR
		**Gene**	******LTBP3******	*LTBP3*	*LTBP3*	*LTBP3*	*LTBP3*	*LTBP3*	*LTBP3*	*LTBP3*
		**Exon**	1/16	1/16	1/16	14	14	splice site acceptor before exon 12		splice site acceptor before exon 21
		**DNA**	c.132delG; c.2248G>T	c.132delG; c.2248G>T	c.132delG; c.2248G>T	c.2033_2041delinsCTT	c.2033_2041delinsCTT	c.1721-2A>G	c.3153_3154del; c.689_690del	c.2894-2A>G
		**Protein**	p.Pro45Argfs*25; p.Glu750*	p.Pro45Argfs*25; p.Glu750*	p.Pro45Argfs*25; p.Glu750*	p.Asn678_Gly681delinsThrCys	p.Asn678_Gly681delinsThrCys	p.?	p.Cys1051*; p.Val230Alafs*16	p.(Ala965Glyfs*38)

**Legend**: M male, F female, + presence, − absence, ± equivocal, NA not available, AD autosomal dominant, AR autosomal recessive, AR* autosomal recessive compound heterozygous. ^a–g^, each letter represents individuals from the same family.

## Data Availability

Identified variant in *LTBP3* gene has been submitted to Global Variome Shared LOVD and it can be accessed using the url: https://databases.lovd.nl/shared/variants/LTBP3?search_position_c_start=2894&search_position_c_start_intron=-2&search_position_c_end=2894&search_position_c_end_intron=-2&search_vot_clean_dna_change=%3D%22c.2894-2A%3EG%22&search_transcriptid=00011553, accessed on 4 August 2021.

## Supplementary Materials

The following is available online at https://www.mdpi.com/article/10.3390/genes12091406/s1, Supplementary Table S1. Whole exome sequencing data output.
