# Supplementary material for: A Rare Case of Brachyolmia with Amelogenesis Imperfecta Caused by a New Pathogenic Splicing Variant in LTBP3
Source: Genes (Basel). 2021 Sep 12;12(9):1406. doi: 10.3390/genes12091406 (PMC8470690; doi:10.3390/genes12091406)
Supplement: Supplementary file 1 [file genes-12-01406-s001.zip › genes-1354874-supplementary.pdf]

Supplementary Table S1. WES data output.

	Data
Target regions coverage, 2x[1]	98.0%
Target regions coverage, 10x[1]	96.5%
Target regions coverage, 20x[1]	94.6%
Average sequencing depth on target[1]	86x
Number of variants with predicted functional effect	12,416
Novel, clinically associated, and unknown/low frequency variants[2]	534
Putative disease genes (Autosomal Recessive/X-linked inheritance)[3]	13[4]
Candidate genes (Autosomal Recessive/X-linked inheritance)	1, *LTBP3*

[1] Referred to Nimblegen SeqCap V.2 (Roche).

[2] MAF <1% in gnomAD v.2.1 database, and frequency <1% in our *in-house* database.

[3] Filtering retained genes with functionally relevant variants by excluding variants predicted as benign by CADD (scaled score <20) and M-CAP algorithms or benign/likely benign by interVar.

[4] *ATP10B* (c.1381G>T, p.Ala461Ser), *CLMN* (c.472C>G, p.Pro158Ala), *COL12A1* (c.1694G>A, p.Arg565Lys), *EDA* (c.1051G>A, p.Val351Ile), *ERN1* (c.1478_1486delAGCAGCAGC, p.Gln493_Gln495del), *KBTBD13* (c.376G>A, p.Ala126Thr), *LTBP3* (c.2894-2A>G), *NSD1* (c.1678G>A, p.Gly560Arg), *PSIP1* (c.1358C>T, p.Ala453Val), *RAB3GAP1* (c.1211A>T, p.Asn404Ile), *SPACA1* (c.437G>A, p.Arg146His), *TPH1* (c.1154A>T, p.Lys385Met), *XBP1* (c.85G>A, p.Gly29Arg).
